# Insights into the Palladium(II)-Catalyzed Wacker-Type Oxidation of Styrene with Hydrogen Peroxide and *tert*-Butyl Hydroperoxide

**DOI:** 10.1021/acscatal.3c05630

**Published:** 2024-01-16

**Authors:** Manting Mu, Katherine L. Walker, Goar Sánchez-Sanz, Robert M. Waymouth, Cristina Trujillo, Mark J. Muldoon, Max García-Melchor

**Affiliations:** † School of Chemistry, Trinity College Dublin, College Green, Dublin 2 Dublin, Ireland; ‡ Department of Chemistry, Stanford University, Stanford, California 94305, United States; § Research IT, The University of Manchester, Oxford Road, Manchester M13 9PL, U.K.; ∥ School of Chemistry and Chemical Engineering, Queen’s University Belfast, Belfast BT71NN, U.K.

**Keywords:** Wacker oxidation, hydride, proton shuttle, density functional theory, palladium, enol−enolate, reaction mechanisms, microkinetic modeling

## Abstract

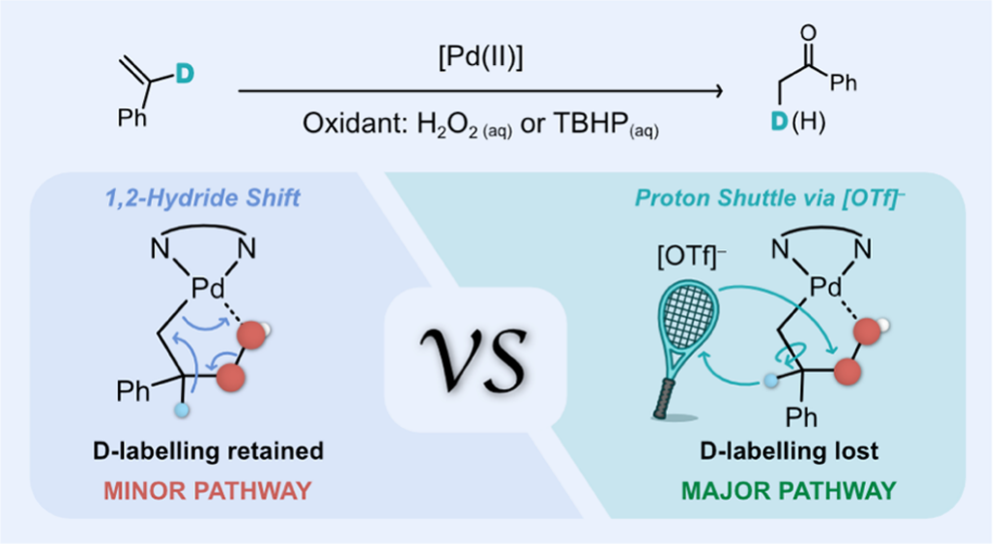

Wacker oxidations are ubiquitous in the direct synthesis of carbonyl compounds from alkenes. While the reaction mechanism has been widely studied under aerobic conditions, much less is known about such processes promoted with peroxides. Here, we report an exhaustive mechanistic investigation of the Wacker oxidation of styrene using hydrogen peroxide (H_2_O_2_) and *tert*-butyl hydroperoxide (TBHP) as oxidants by combining density functional theory and microkinetic modeling. Our results with H_2_O_2_ uncover a previously unreported reaction pathway that involves an intermolecular proton transfer assisted by the counterion [OTf]^−^ present in the reaction media. Furthermore, we show that when TBHP is used as an oxidant instead of H_2_O_2_, the reaction mechanism switches to an intramolecular protonation sourced by the HO*t*Bu moiety generated in situ. Importantly, these two mechanisms are predicted to outcompete the 1,2-hydride shift pathway previously proposed in the literature and account for the level of D incorporation in the product observed in labeling experiments with α-d-styrene and D_2_O_2_. We envision that these insights will pave the way for the rational design of more efficient catalysts for the industrial production of chemical feedstocks and fine chemicals.

## Introduction

The Wacker process, which involves the aerobic oxidation of ethylene to acetaldehyde catalyzed by Pd and Cu salts,^1^ is a textbook example of industrial homogeneous catalysis. Its mechanism has been studied in great depth for decades,^2^ and modified methods that enable the oxidation of larger alkenes have proven useful in organic synthesis.^3−8^ The majority of these experimental and computational studies have been carried out in aerobic conditions,^9−11^ although it is also possible for Pd(II) catalysts to use peroxides to oxidize alkenes to ketones. The use of H_2_O_2_ for the Wacker oxidation of ethylene was documented in 1960,^12^ followed by reports in 1980 wherein H_2_O_2_ or *tert*-butyl hydroperoxide (TBHP) was used for the oxidation of larger alkenes.^13−15^ However, there are far fewer studies of peroxide-mediated Wacker reactions in comparison to the aerobic processes, and hence, there is still scope for developing more efficient systems and a better understanding of these reactions.

Significant strides were made when Sigman and co-workers developed synthetic methods using TBHP as an oxidant. Their approach consisted of reacting a quinoline-2-oxazoline (Quinox) ligand with PdCl_2_ to generate, in situ, a dicationic complex in the presence of Ag[SbF_6_], allowing the oxidation of a range of alkenes to their desired ketones in excellent yields.^15−19^ Furthermore, this TBHP catalyst system often outperforms aerobic methods and has been applied in target-orientated synthesis studies.^20−23^

Nonetheless, fewer studies have been reported with H_2_O_2_-mediated systems, which prompted us to begin exploring this area. Using H_2_O_2_ as an oxidant, we found that the dicationic Pd(II) complex, [(PBO)Pd(NCMe)_2_][OTf]_2_ (PBO = 2-(pyridin-2-yl)benzoxazole and OTf = trifluoromethanesulfonate), could act as a catalyst with good selectivity for styrenyl substrates.^24^ The Sigman group had carried out mechanistic studies on their TBHP system and proposed a detailed catalytic cycle.^25^ Their mechanism was in good agreement with previous proposals by Mimoun and co-workers, who had suggested a similar mechanism for both H_2_O_2_ and TBHP-mediated reactions.^14,15^ However, we felt that there were likely key differences as the two oxidants resulted in different catalyst performances. This led us to carry out kinetic, isotope labeling, and high-resolution mass spectrometry studies to try to understand these systems in more detail.^26^ Interestingly, our findings revealed that there was more than one reaction pathway leading to the desired product when H_2_O_2_ was used as the oxidant, as we detail below.

**Scheme 1:**
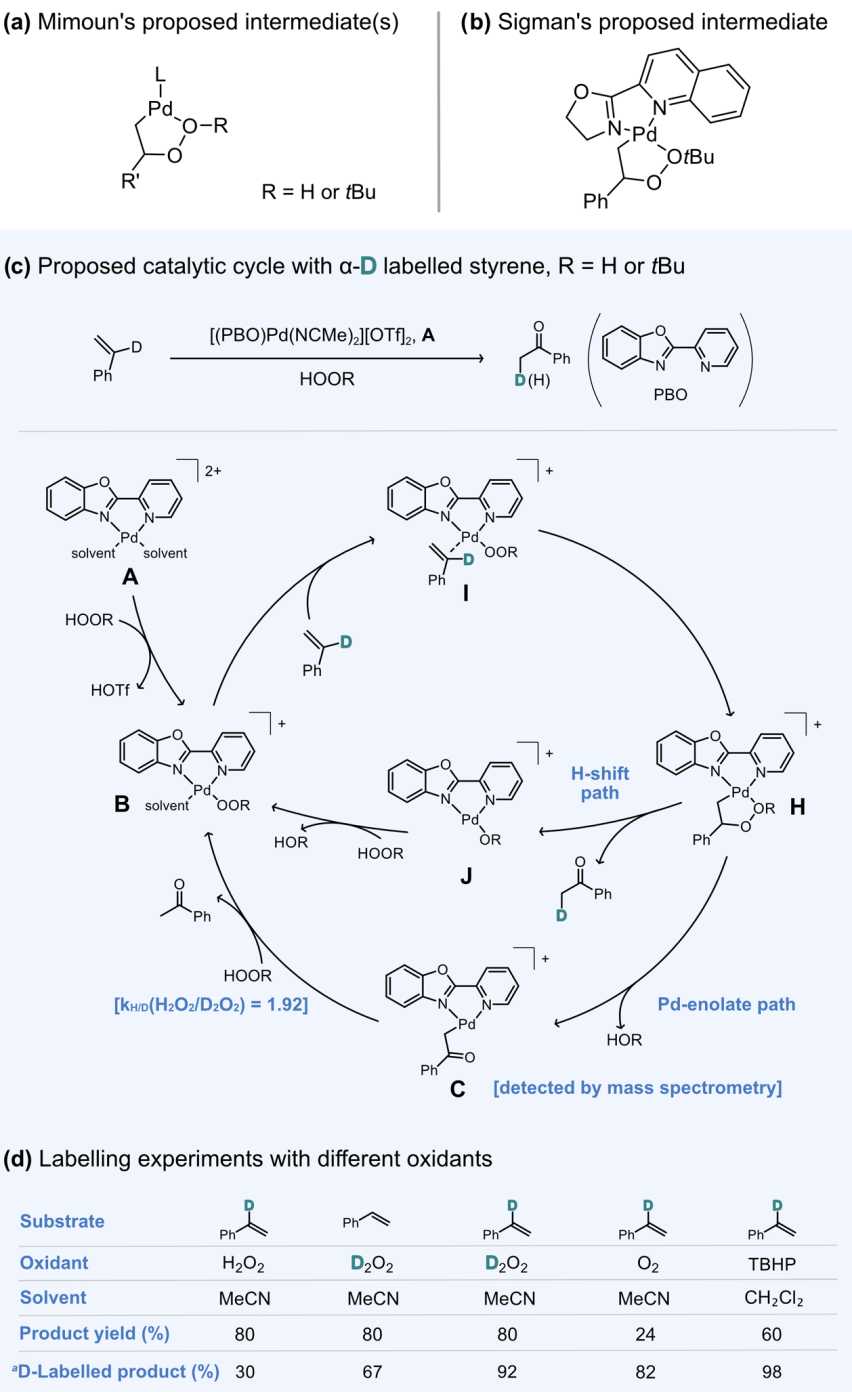
Reaction Intermediates for the Wacker Oxidation of Alkenes to Form Carbonyl Compounds Proposed by the (a) Mimoun and (b) Sigman Groups. (c) Previously Proposed Catalytic Cycle for the Wacker Oxidation of Styrene with H_2_O_2_ and TBHP as Oxidants. (d) Summary of Reported D-Labeling Studies and Experiments with Different Oxidants^26^ The percentage of D-labeled product is calculated out of the total product yield.

In situ mass spectrometry studies identified species that confirmed the previous suggestions by both the Mimoun and Sigman groups that palladacyclic (alkylperoxide) complexes are key intermediates in the Wacker oxidation with both H_2_O_2_ and TBHP (Scheme 1a,b). However, labeling studies made it clear that H_2_O_2_ and TBHP-mediated reactions did not proceed via identical pathways. In particular, experiments with α-d-styrene indicated that acetophenone was not produced solely via a 1,2-hydride transfer, as when H_2_O_2_ was the oxidant, only 30% of the product was deuterated (Scheme 1c,d). Control experiments with water and without H_2_O_2_ did result in the formation of acetophenone, although in a much lower yield compared to when H_2_O_2_ was present (ca. 24%, Scheme 1d). This is a consequence of H_2_O acting as the nucleophile and the reaction proceeded via an aerobic cycle (not shown in Scheme 1). Such a pathway did not account for the low D incorporation observed, as it was found that these conditions with α-d-styrene led to 82% D incorporation in the product.

On the other hand, reactivity studies with TBHP, using both the PBO catalyst and Sigman’s Quinox catalyst, revealed that acetophenone was mainly produced via the hydride shift pathway. In addition, experiments using ^18^O-labeled H_2_O_2_ confirmed that the peroxide pathway dominated. Altogether, these observations implied that H_2_O_2_-mediated Wacker oxidation was proceeding via a different reaction mechanism.

Further insights were obtained by mass spectrometry studies, which provided evidence for a palladium enolate intermediate (**C**, Scheme 1c) that accounted for the loss of the α-hydrogen. In light of these findings, we proposed the enolate species to be a key intermediate in the predominant pathway, whereby acetophenone is liberated via protonation by H_2_O_2_, with the concomitant regeneration of the active palladium peroxide species. Although this hypothesis was supported by studies with D_2_O_2_, the details of the formation of the palladium enolate intermediate are unknown. Furthermore, it is unclear why the mechanism would change when the reaction is carried out with H_2_O_2_ vs TBHP.

Herein, we present a thorough mechanistic investigation of the Wacker oxidation of styrene using H_2_O_2_ and TBHP as oxidants. Density functional theory (DFT) calculations reveal the existence of two competing pathways in the reaction with H_2_O_2_, namely, the previously proposed 1,2-hydride shift, and a newly uncovered proton shuttle mechanism assisted by the [OTf]^−^ counterion that involves the formation of a stable C-bound Pd-enolate intermediate. Microkinetic modeling in combination with reported D-labeling experiments indicates that the proton shuttle pathway is the dominant mechanism. Furthermore, DFT calculations show that when TBHP is used as an oxidant, the reaction proceeds via an intramolecular protonation pathway instead of the intermolecular protonation predicted with H_2_O_2_ or the previously proposed 1,2-hydride shift pathway.^27^ We believe that these new insights will inspire and support the development of more efficient systems for Wacker-type oxidation reactions. The oxidation of an alkene to a ketone is a reaction that is applied to the production of both commodity chemicals and fine chemicals, such as active pharmaceutical ingredients, which still require more effective and selective catalysts for these industrial processes. A deeper understanding of catalytic methods is vital for the rational development of better catalysts.

## Results and Discussion

**Figure 1:**
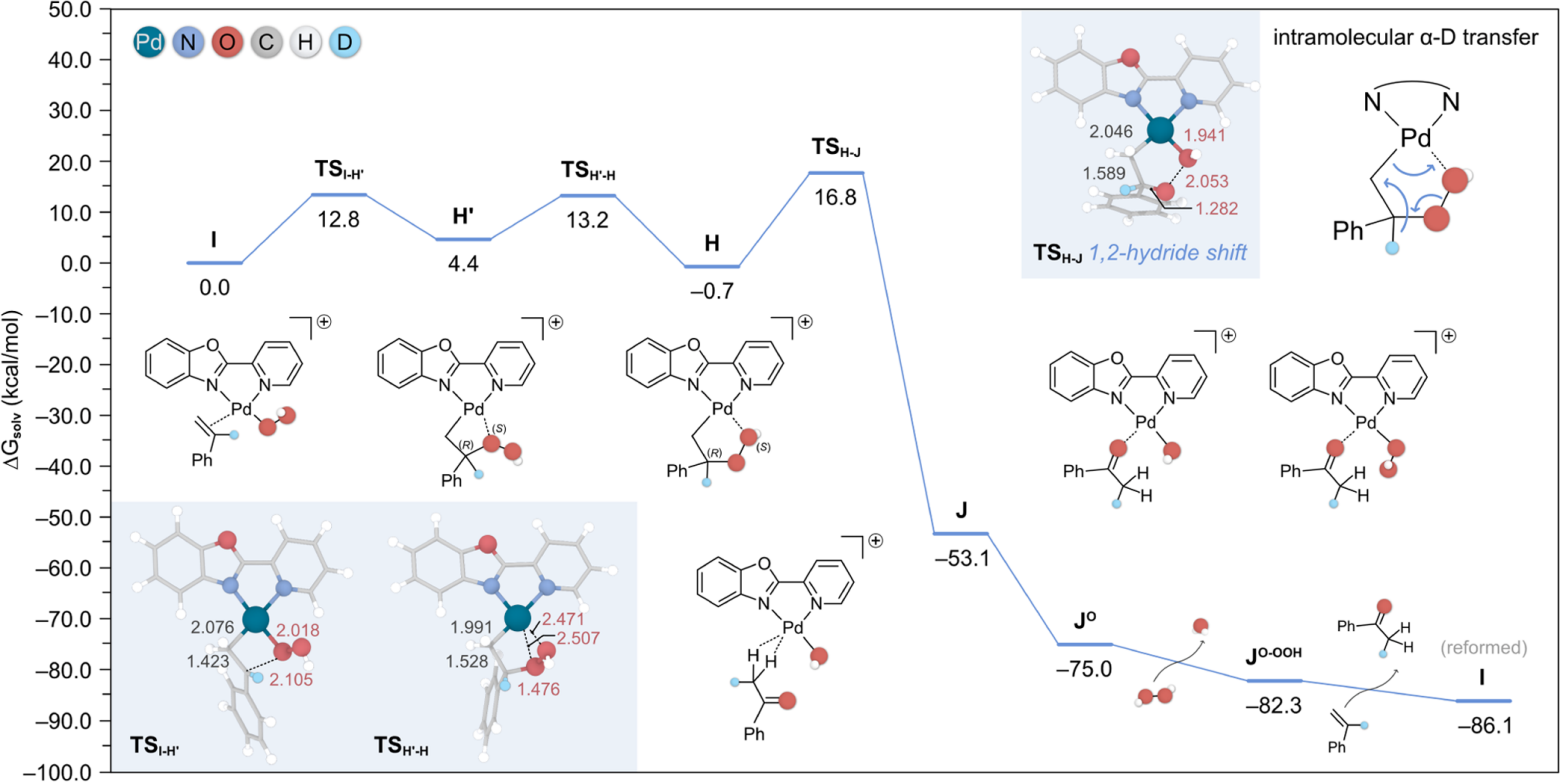
Gibbs energy profile (in kcal/mol) calculated at 1 atm and 298.15 K for the 1,2-hydride shift pathway of the Wacker oxidation of α-d-styrene using H_2_O_2_ as oxidant. The optimized transition state structures are depicted as insets with the relevant bond distances shown in Å. For clarity, the deuterated α-hydrogen atom is highlighted as a light blue sphere.

To address the above open questions around the Wacker process, we performed a detailed DFT investigation at the ωB97XD level (see the Supporting Information (SI) for details) of the oxidation of styrene catalyzed by [(PBO)Pd(NCMe)_2_][OTf]_2_ with H_2_O_2_ and TBHP as oxidants. Previous mass spectrometry studies have shown that the formation of the Pd-peroxide complex (**B**) from the starting reagents (**A**) is kinetically fast (Scheme 1c),^26^ which our DFT calculations confirmed with a predicted Gibbs energy of −22.9 kcal/mol using H_2_O_2_ as oxidant and DCM as solvent (see the SI for a detailed discussion on solvent effects). We then proceeded to model the coordination of styrene to yield intermediate **I**. From this species, the reaction mechanism was mapped out with the lowest energy profile depicted in Figure 1.

Once complex **I** is formed, the insertion of the Pd-bound O atom into the vinyl-C of styrene affords the four-membered palladacycle **H’** via a transition state (**TS**_**I–H’**_) with an energy barrier of 12.8 kcal/mol, indicative of a fast chemical process. Notably, the formation of the C–O bond in this step causes some rearrangement around the Pd center and introduces two chiral centers at the vinyl-C and the Pd-bound O atoms. Similar pathways featuring other stereoisomers of **H’** were also considered (Table S1), but they were overall found to be less favorable than the (*RS*)-enantiomeric path shown in Figure 1. Further rearrangement and a change in coordination to establish a Pd–OH adduct upon rotation of the OOH moiety give complex **H**, which is the same palladacycle proposed in our previous experimental studies (Scheme 1c). The formation of this 5-membered ring species is exergonic by −5.1 kcal/mol, which can be attributed to the release of steric strain compared to the 4-membered structure in **H’**. Subsequently, intermediate **H** undergoes a 1,2-hydride shift that triggers the O–O bond cleavage to form the acetophenone product (**J**) in a thermodynamically favorable process by −52.4 kcal/mol. This involves a transition state (**TS**_**H–J**_) with a relative energy barrier of 17.5 kcal/mol, rendering this step rate-limiting. We also note that the transfer of the hydride from vinyl-C to terminal C results in acetophenone with the internal α-H atom retained. Hence, this reaction pathway is expected to produce D-labeled acetophenone when α-d-styrene is used as substrate.

The acetophenone produced in complex **J** can then be released in solution or rebound to Pd through the O atom to form the isomeric form **J**^**O**^, which calculations predict to be more stable by a factor of −21.9 kcal/mol. Next, the oxidant H_2_O_2_ can be deprotonated by the Pd–OH species to eliminate a H_2_O molecule and yield the Pd–OOH intermediate (**J**^**O–OOH**^) in an exergonic process by −7.3 kcal/mol. Finally, a new styrene molecule displaces the acetophenone product to regenerate starting complex **I** and close the catalytic cycle.

**Figure 2:**
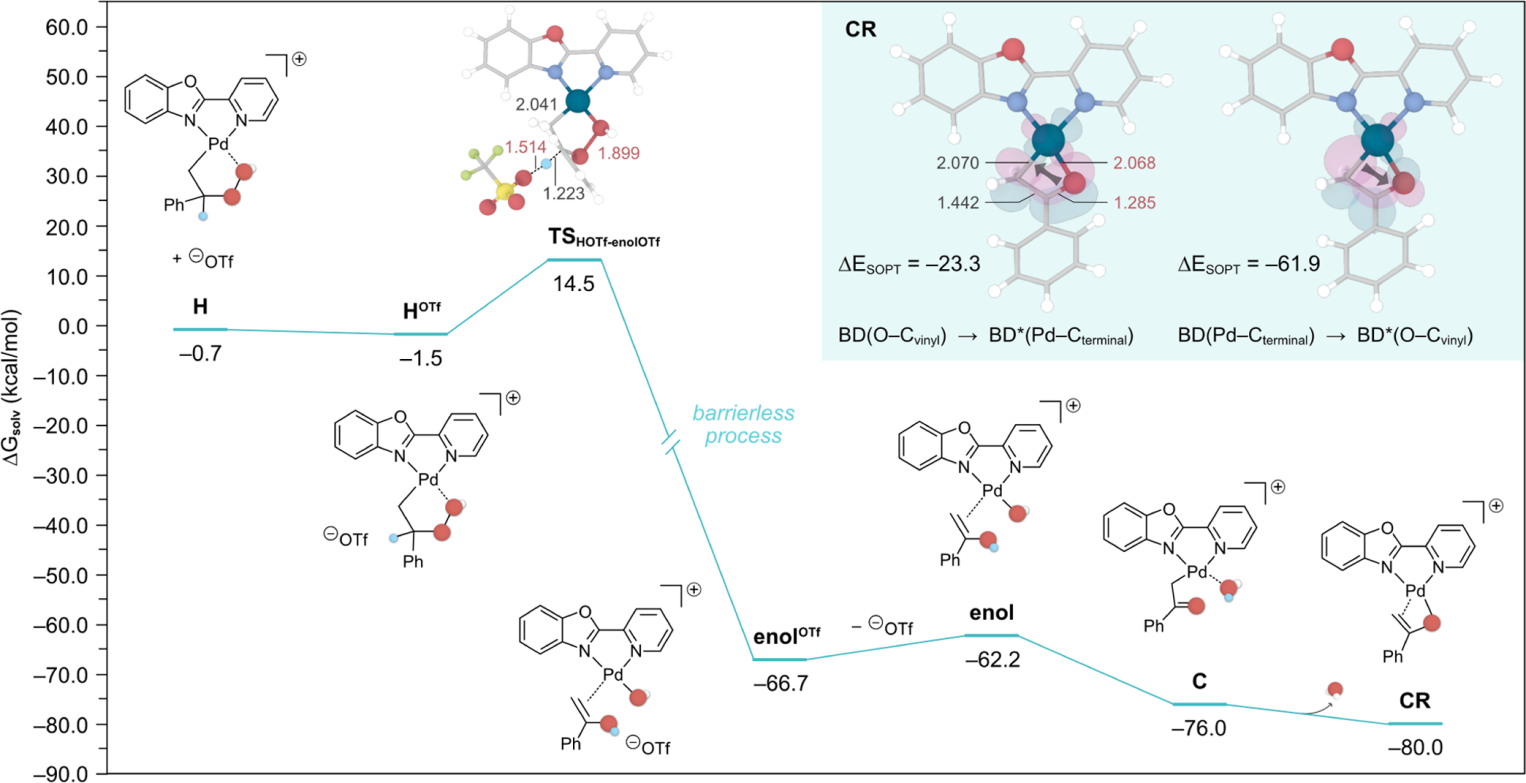
Gibbs energy profile (in kcal/mol) calculated at 1 atm and 298.15 K for the proton shuttle pathway in the Wacker oxidation of α-d-styrene using H_2_O_2_ as oxidant. The optimized transition state structure is depicted as an inset with the relevant bond distances shown in Å. For clarity, the deuterated α-hydrogen atom is highlighted as a light blue sphere. Atom color code is the same as in Figure 1. Inset (top right): natural bond orbital analysis (isovalue = 0.05 au) of intermediate **CR**. The donor–acceptor interaction energies (in kcal/mol) calculated via second-order perturbation theory (SOPT), as well as the natural orbitals involved, are also provided. BD and BD* denote bonding and antibonding orbitals, respectively.

In an attempt to account for the low yield of the D-labeled product obtained in experiments with α-d-styrene, we explored the possibility that the α-hydrogen in **H** (Figure 1, −0.7 kcal/mol) could be abstracted by the triflate counteranion present in solution. To examine this hypothesis, we modeled the corresponding interacting ion-pair structure (**H**^**OTf**^), shown in Figure 2, which is predicted to be more stable by −0.8 kcal/mol compared to **H**. From this intermediate, we could then locate a transition state (**TS**_**HOTf-enolOTf**_) whereby the triflate anion deprotonates the α-H atom, with an energy barrier of 16.0 kcal/mol. Importantly, this barrier is lower than that found for the 1,2-hydride pathway (i.e., 18.3 kcal/mol, relative to the lowest preceding intermediate, **H**^**OTf**^), rendering this alternative mechanism more feasible under the experimental conditions.

After modestly displacing (by a factor of 0.5) the coordinates of the atoms involved in **TS**_**HOTf-enolOTf**_ along the eigenvector associated with the imaginary frequency and subsequently optimizing the structure to an energy minimum, we observed the direct transfer of a proton from triflic acid to the C-bound oxygen, resulting in the formation of a Pd-enol intermediate (**enol**^**OTf**^). This observation suggests that the associated process entails a negligible barrier. This step is followed by the dissociation of the triflate anion and the rotation of the O–H bond, resulting in the transfer of the proton to afford the Pd-enolate species **C** and a coordinated water in an exergonic process by −13.8 kcal/mol. Further dissociation of the water molecule leads to the isomeric enolate species **CR**, which is also thermodynamically driven and explains the experimental observation of this intermediate.

The possibility of water acting as proton shuttle instead of the triflate anion was also investigated by modeling the corresponding transition state with one and two water molecules. In both cases, the calculated barriers were found to be slightly higher than with triflate (Scheme S1), in line with the relative acidity of these species. Overall, these results highlight the noninnocent role of the triflate counteranion by enabling a competitive pathway that explains both the experimental observation of a Pd-enolate species and the low D incorporation in the acetophenone product. We also note that the role of triflate in facilitating proton transfer steps has also been reported in other reactions.^28,29^

To understand the remarkable stability of Pd-enolate **CR**, we next performed a natural bond orbital (NBO) analysis, the results of which are summarized in the inset of Figure 2. This investigation identified two main interactions, namely, the donation from a bonding (BD) orbital of Pd–C_terminal_ to an O–C_vinyl_ antibonding (BD*) orbital, and the backdonation from a π(O–C_vinyl_) BD orbital to a Pd–C_terminal_ BD* orbital, as detailed in Table S2. These donor–acceptor interactions were further quantified via second-order perturbation theory (SOPT) to be −61.9 and −23.3 kcal/mol, respectively. These interactions are further emphasized by the short Pd–C and Pd–O interatomic distances (i.e., 2.070 and 2.068 Å, respectively) compared to the sum of their covalent radii (Pd–C = 2.15 Å and Pd–O = 2.05 Å).^30^ In light of this, and the fact that the calculated C–C and C–O bond distances (Figure 2) are in between the single and double bonds in acetophenone and its enol form, respectively (i.e., C–C = 1.507 > 1.442 > 1.335 Å; C–O = 1.371 > 1.285 > 1.225 Å), we deduce that the enolate character is in resonance between the C and O atoms. This effect may explain the noticeable stability of this **CR** intermediate and, therefore, its experimental observation.

**Figure 3:**
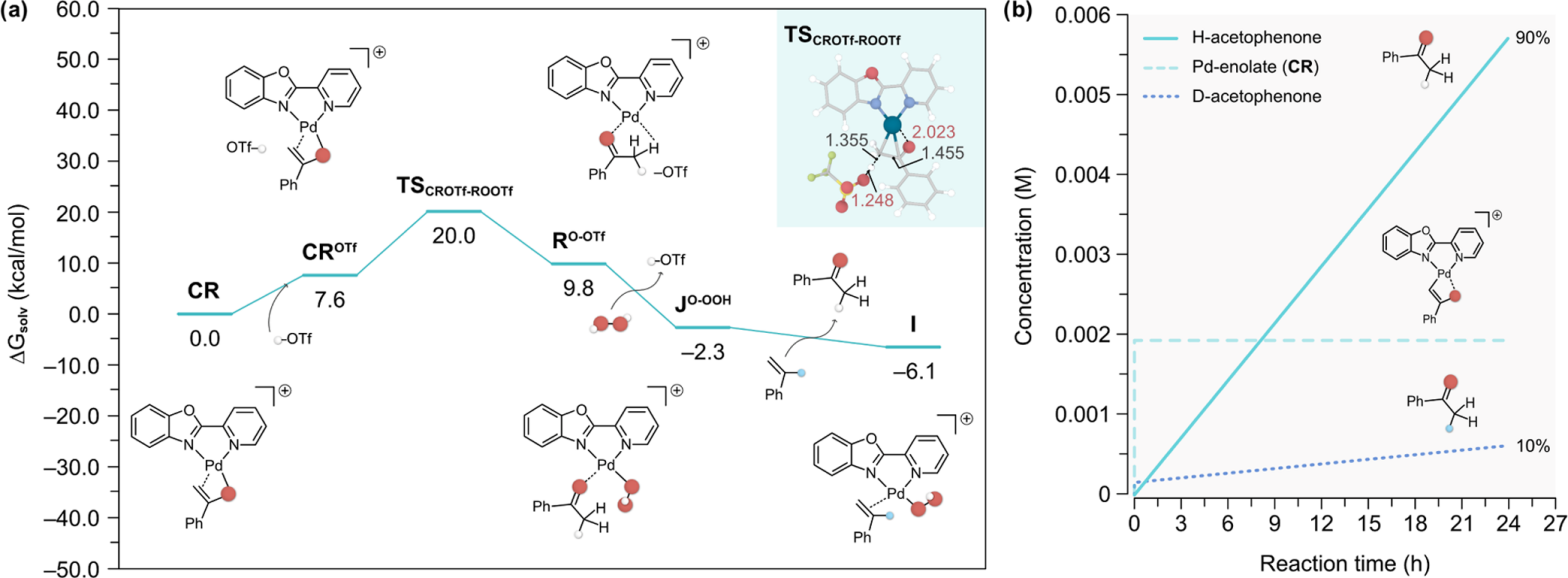
(a) Gibbs energy profile (in kcal/mol) calculated at 1 atm and 298.15 K for the proton shuttle pathway in the Wacker oxidation of α-d-styrene using H_2_O_2_ as the oxidant, from the intermediate **CR** to **I**. The optimized transition state structure is depicted as an inset with the relevant bond distances shown in Å. Atom color code is the same as in Figure 1. (b) Concentration profile obtained via microkinetic modeling using DFT-calculated data (see the Supporting Information for details).

After the formation of the Pd-enolate **CR**, the reaction progresses to regenerate the starting species **I** through a series of elementary steps, as depicted in Figure 3a, which we detail in the following. The terminal C of the enolate is protonated by the triflic acid—generated at the start of the reaction upon deprotonation of H_2_O_2_ in going from intermediate **A** to **B** (Scheme 1c)—to yield an intermediate (**R**^**O-OTf**^) wherein acetophenone is interacting with the Pd center (Pd–O = 2.006 Å; Pd–H = 1.978 Å). This protonation step involves a transition state (**TS**_**CROTf-ROOTf**_) with a feasible energy barrier of 20.0 kcal/mol, which highlights the important role of triflic acid generated in situ as reported by Hintermann and co-workers in catalytic processes involving metal triflates.^31^ Alternative proton transfers assisted by both H_2_O and H_2_O_2_ instead of triflic acid were also explored. Calculations confirmed that these reagents can act as proton donors under the experimental conditions of this work with slightly higher barriers of 25.3 and 26.4 kcal/mol, respectively (Schemes S2 and S3).

Overall, this protonation of the Pd-bound enolate was found to be the rate-limiting step (**TS**_**CROTf-ROOTf**_) of the entire process. It is important to note that while this barrier (20.0 kcal/mol, Figure 3) is higher than that of the 1,2-hydride shift pathway (18.3 kcal/mol, Figure 2), it does not affect the preference for this pathway. This is because the formation of the very stable enolate species **CR** is irreversible, and therefore, the reaction can only progress further via the proton shuttle pathway to give **R**^**O-OTf**^. The triflate anion generated in this step subsequently deprotonates another H_2_O_2_ molecule to yield species **J**^**O-OOH**^ in an exergonic process by –12.1 kcal/mol, followed by the regeneration of starting intermediate **I** through the substitution of acetophenone by styrene.

The dominance of the proton shuttle pathway predicted by DFT calculations is further supported by experimental studies with D_2_O_2_/D_2_O and unlabeled styrene, leading to 67% of D incorporation in the final product (Scheme 1d). This observation, and the fact that 70% of the unlabeled product is obtained with α-d-styrene, demonstrates that D incorporation is mainly sourced by the oxidant D_2_O_2_/D_2_O through the proton shuttle mechanism (Figure 3). Further validation can be obtained by relating the computed rate-limiting transition states to the kinetic isotope effect (KIE) measured experimentally with different deuterated reagents. In particular, experiments with D_2_O_2_/D_2_O are expected to deuterate triflic acid in the process of going from intermediate **A** to **B** (Scheme 1c), which is then involved in the protonation of the enolate via **TS**_**CROTf-ROOTf**_, the rate-limiting step of the overall reaction. Because this step involves the transfer of the D/H atom, a KIE of >1 is expected, in line with the value of 1.92 measured experimentally. On the other hand, when α-d-styrene is used, the reaction rate should not be affected since the α-D/H transfer occurs in the 1,2-hydride shift via **TS**_**H-J**_, which is not rate-limiting. This is consistent with the experimental KIE = 0.97 obtained in these conditions.^26^

To conclusively demonstrate the dominance of the proton shuttle mechanism, we performed microkinetic modeling (MKM) studies using the DFT-calculated energies reported in Figures 1–3 (see the Supporting Information for details). In particular, we modeled the reaction conditions with α-d-styrene and nonlabeled oxidant H_2_O_2_/H_2_O, which experimentally affords 30% of d-acetophenone and 70% of H-acetophenone. This trend was confirmed in the concentration profiles simulated by MKM, shown in Figure 3b, leading to 10% d-acetophenone and 90% H-acetophenone. We note that the lower percentage of D-labeled products compared to experiments may be partially attributed to the fact that our MKM model only considers the DFT-modeled anaerobic pathways, while experiments were run under aerobic conditions.

**Figure 4:**
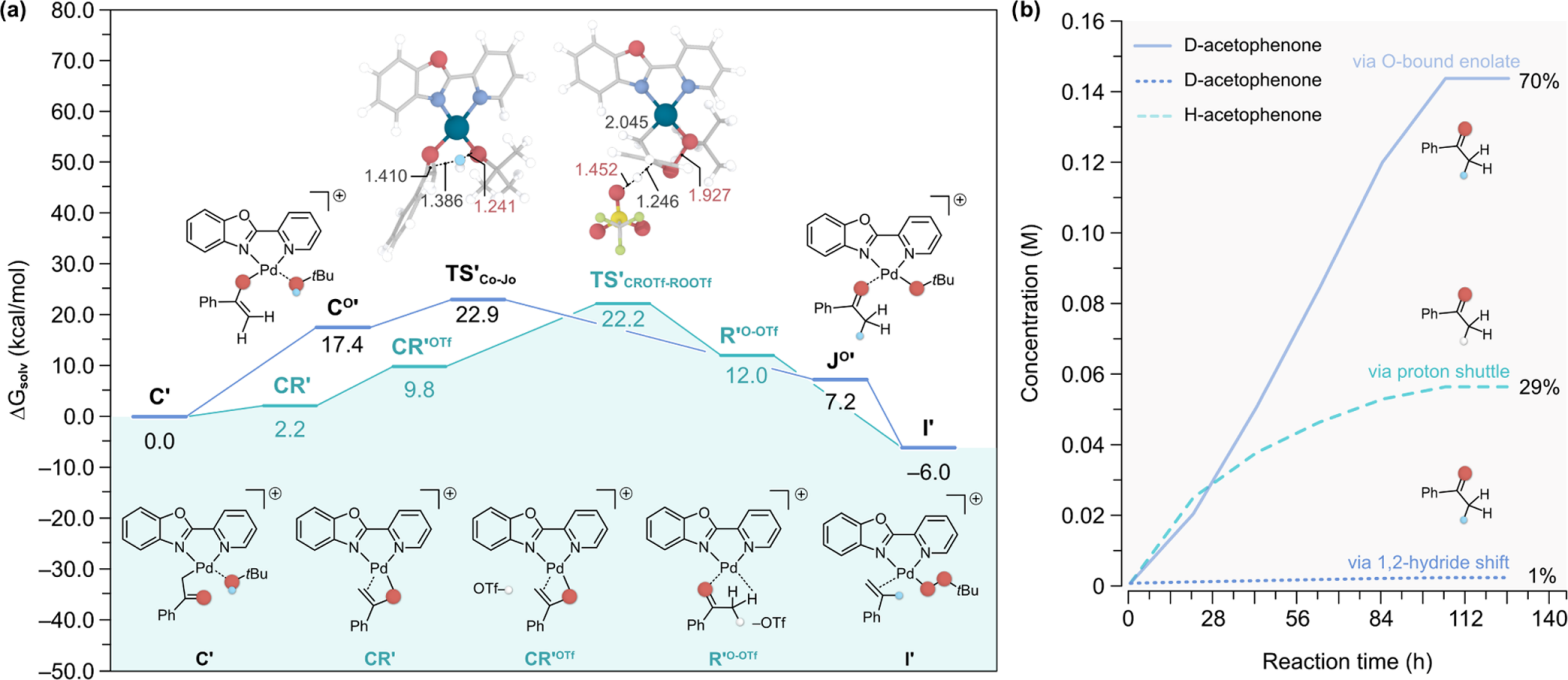
(a) Gibbs energy profile (in kcal/mol) calculated at 1 atm and 298.15 K for the proton shuttle pathway in the Wacker oxidation of α-d-styrene using TBHP as oxidant, from intermediate **C’** to **I’**. The optimized transition state structure is depicted as an inset with the relevant bond distances shown in Å. Atom color code is the same as in Figure 1. (b) Concentration profile obtained via microkinetic modeling using DFT-calculated data (see the Supporting Information for details).

Interestingly, experiments with α-d-styrene and TBHP as oxidant afforded 98% of the D-labeled product compared to 30% with H_2_O_2_ (Scheme 1d), hinting at a mechanistic switch. To shed light on this markedly different reactivity, we computed the 1,2-hydride shift and proton shuttle pathways with styrene and TBHP (Figures S1 and S2). Notably, the proton shuttle mechanism was again found to require a lower energy barrier compared to the generally accepted 1,2-hydride shift pathway (i.e., 18.6 vs 21.8 kcal/mol). However, DFT calculations revealed one key difference between the two oxidants, namely, the TBHP equivalent of the Pd-enolate, **CR’** (Figure 4a), was found to be less stable than the analogous species with H_2_O_2_, **CR**. We attribute this difference to the formation of a water molecule from H_2_O_2_ coordinated to Pd in intermediate **C** (Figure 2), while TBHP yields a HO*t*Bu group in **C’** (Figure 4a). Unlike water, the dissociation of HO*t*Bu is thermodynamically unfavorable by −2.2 kcal/mol. This opens up an alternative reaction mechanism whereby the H atom in HO*t*Bu is transferred to the terminal C of styrene. For this process to occur, the coordination of the Pd-bound enolate shifts from a C (**C’**) to an O-bound enolate (**C**^**O**^**’**). Although the computed transition state for the H transfer via this mechanism (**TS’**_**Co-Jo**_, 22.9 kcal/mol) is slightly higher in energy compared to the one with HOTf (**TS’**_**CROTf-ROOTf**_, 22.2 kcal/mol), this protonation step is first order with respect to the concentration of triflic acid, thus limiting the reaction rate through this pathway. Given the relatively low concentration of HOTf present in solution, we propose that HO*t*Bu acts as the main proton source.

To verify the above hypothesis, we performed MKM simulations with TBHP (Figure 4b), which predict the formation of 71% of d-acetophenone and 29% of H-acetophenone. It is also worth noting that the 1,2-hydride shift pathway is predicted to contribute with a mere 1% compared to 70% via the newly uncovered intramolecular protonation sourced by the HO*t*Bu generated in situ. Overall, these results are in line with the experimental observations that TBHP leads to a higher degree of D-incorporation. In particular, we previously examined the oxidation of α-d-styrene with TBHP using [(PBO)Pd(NCMe)_2_][OTf]_2_ and Sigman’s (Quinox)PdCl_2_/Ag[SbF_6_] catalyst, obtaining d-acetophenone with 98% incorporation.^26^ While this level of incorporation is higher than that predicted by our MKM simulations, it is worth bearing in mind that experimental studies also have variation and additional factors. For example, an earlier study by Sigman et al. oxidized α-d-styrene with TBHP using a Pd catalyst with an *N*-heterocyclic carbene ligand, producing d-acetophenone with 81% incorporation.^32^ In addition, we note that in all experimental studies that investigate the oxidation of styrenes, it is found that the reaction is not 100% selective and a number of other oxidation products can be produced.^16,26,27,32^ While these products are not produced via the same mechanism, it is possible that they have an influence on the results obtained in experimental studies.

In summary, herein we report a thorough mechanistic investigation of the Wacker oxidation of styrene with H_2_O_2_ and TBHP as oxidants by combining DFT calculations and MKM. We have uncovered a new reaction pathway that involves the triflate counteranion as a proton shuttle, as well as exploring the widely known 1,2-hydride shift mechanism. Our findings show that the proton shuttle mechanism dominates under the reaction conditions of this work and explains the trends observed in the D-labeling experiments with α-d-styrene and D_2_O_2_/D_2_O. In addition, we shed light into the different degrees of D incorporation found in experiments using H_2_O_2_ and TBHP as oxidants. This is attributed to a mechanistic switch for the reaction with TBHP, which transitions from an intermolecular proton transfer (assisted by triflic acid) to an intramolecular protonation sourced from HO*t*Bu generated in situ.

Overall, this work highlights the importance of the chemical oxidant and the noninnocent role of the counteranion in assisting H atom transfer steps. Future research could involve the optimization of the electronic and steric properties of the ligand as well as the reaction conditions (e.g., pH, solvent). Recent experimental studies by us and others have highlighted that these factors can have a significant impact on catalyst performance.^27,33,34^ Understanding these factors in-depth will contribute to the development of more effective catalytic methods for this important type of oxidation reaction.

## Supporting Information Available

Computational and experimental details as well as any additional results, and all of the DFT-optimized structures reported in this work are openly accessible from the ioChem-BD repository available at the following link: 10.19061/iochem-bd-6-314 (PDF)
