# Supplementary material for: Insights into the Palladium(II)-Catalyzed Wacker-Type Oxidation of Styrene with Hydrogen Peroxide and tert-Butyl Hydroperoxide
Source: ACS Catal. 2024 Jan 16;14(3):1567–74. doi: 10.1021/acscatal.3c05630 (PMC10845106; doi:10.1021/acscatal.3c05630)
Supplement: Supplementary file 1 — cs3c05630_si_001.pdf [file cs3c05630_si_001.pdf]

Supporting Information for:

Insights on the palladium(II)-catalyzed Wacker-type oxidation of styrene with hydrogen peroxide and *tert*-butyl hydroperoxide

Manting Mu,[†] Katherine L. Walker,[‡] Goar Sánchez-Sanz,[¶] Robert M. Waymouth,[‡,*] Cristina Trujillo,[†,§,*] Mark J. Muldoon,[||,*] and Max García-Melchor[†,*]

[†]School of Chemistry, Trinity College Dublin, College Green, Dublin 2, Dublin, Ireland.

[‡]Department of Chemistry, Stanford University, Stanford, California 94305, United States.

[¶]Research IT, The University of Manchester, Oxford Road, Manchester, M13 9PL, UK.

[§]Department of Chemistry, The University of Manchester, Oxford Road, Manchester, M13 9PL, UK.

[||]School of Chemistry and Chemical Engineering, Queen's University Belfast, Belfast, BT71NN, United Kingdom.

E-mails of corresponding authors:

Robert M. Waymouth: waymouth@stanford.edu

Cristina Trujillo: cristina.trujillodelvalle@manchester.ac.uk

Mark J. Muldoon: m.j.muldoon@qub.ac.uk

Max García-Melchor: garciamm@tcd.ie

Table of Contents

Computational details 2

Stereoisomers of $TS_{I-H'}$, H', $TS_{H'-H}$, H and TS_{H-J} 3

Alternative pathways for water acting as proton shuttle 4

Alternative proton transfer pathways of Pd-enolate with H_2O and H_2O_2 as proton sources 5

Natural bond orbital analysis of the Pd-enolate intermediate 6

Microkinetic studies of the Wacker oxidation of styrene with H_2O_2 as the oxidant 7

Competing pathways for Wacker oxidation of styrene involving TBHP as the oxidant 9

Microkinetic studies of the Wacker oxidation of styrene with TBHP as the oxidant..........11

Cartesian coordinates and energies of the modelled structures..........13

References 14

Computational details

DFT calculations reported in this work were performed by means of the hybrid ωB97XD[1] functional implemented in the Gaussian09 program package.[2] The Stuttgart-Dresden (SDD) effective core potential[3] was used to describe the inner electrons of the Pd center, while its associated double-ζ basis set was employed to describe the valence electrons. Additionally, an *f*-polarization (exponent = 1.472)[4] gaussian function was added to describe this metal. For C and H atoms, the 6-31G(d,p) basis set was employed, whereas the more electronegative N, O, S and F atoms were described with the same basis set including a diffuse function, *i.e.* 6-31+G(d,p).[5-8]

The structures of the reactants, intermediates, and transition states were fully optimized in solvent (see below) without any symmetry constraint. The nature of the stationary points in the potential energy surface, whether minima or saddle points, were characterized via vibrational frequency calculations. Transition states were confirmed to have one imaginary frequency and to relate the expected reactants/products.

Solvent effects (CH_2Cl_2, ε = 8.930) were included in the geometry optimizations through the continuum solvation model based on density (SMD),[9] implemented in Gaussian09.

We note that experimental studies[10] were reported in different solvents – MeCN for H_2O_2 and CH_2Cl_2 for TBHP. To ensure a meaningful comparison, in this work we modeled both reactions using CH_2Cl_2 as the solvent in our initial DFT calculations. In anticipation of potential solvent effects, we conducted benchmark calculations by reoptimizing the reaction intermediates **I**, **H'** and **H** in MeCN, finding energy differences of less than 1 kcal/mol. Furthermore, we reoptimized $\mathbf{TS_{HOTf\text{-}enolOTF}}$ (Figure 1) and $\mathbf{TS1_{HH2O\text{-}enolH2O}}$ (Scheme S1) using MeCN as the solvent for the Wacker process with H_2O_2 as oxidant. The resulting energy barriers are +14.6 and +23.4 kcal/mol, respectively, compared to +14.5 and +20.5 kcal/mol in CH_2Cl_2. Importantly, these results are quite similar and do not alter the overall conclusions of our work.

Natural bonding orbital (NBO) analysis was performed using the converged wavefunctions and structures with the NBO 7.0 software.[11]

Stereoisomers of $\mathbf{TS_{I\text{-}H'}}$, H’, $\mathbf{TS_{H'\text{-}H}}$, H and $\mathbf{TS_{H\text{-}J}}$

Table S1. Relative Gibbs energies (in kcal/mol) of the stereoisomers *RR, RS, SS* and *SR* for the species **$TS_{I\text{-}H'}$**, **H’**, **$TS_{H'\text{-}H}$**, **H** and **$TS_{H\text{-}J}$**. Energies are referenced to the intermediate **I**, as shown in Figure 1. Based on the energy of the rate determining step (**$TS_{H\text{-}J}$**) we highlight in bold the energies of the species involved in the most favorable pathway.

	RR	***RS***	***SS***	***SR***
**$TS_{I\text{-}H'}$**	12.8	-	11.8	-
**H’**	4.4	**4.4**	6.1	6.1
**$TS_{H'\text{-}H}$**	12.0	**13.2**	13.7	13.5
**H**	–1.8	**–0.7**	–1.0	–2.9
**$TS_{H\text{-}J}$**	17.7	**16.8**	17.2	17.7

Alternative pathways for water acting as proton shuttle

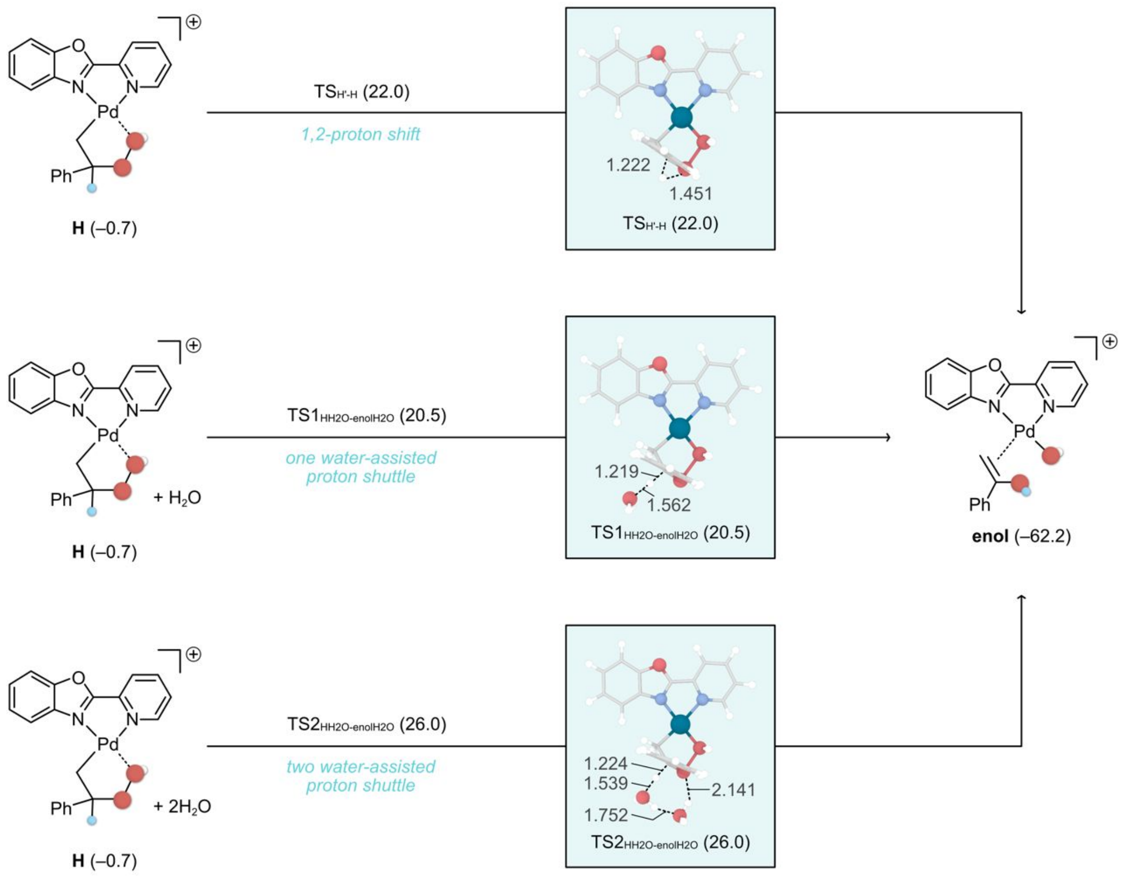

Scheme S1. Alternative pathways computed from **H** to form **enol** assisted via 0, 1, or 2 molecules of water. Gibbs energies are reported at the experimental conditions in kcal/mol. The energies of the intermediates are referenced to **I** (Figure 1), while the transition state energies are referenced to most stable intermediate **H**.

Alternative proton transfer pathways of Pd-enolate with H_2O and H_2O_2 as proton sources

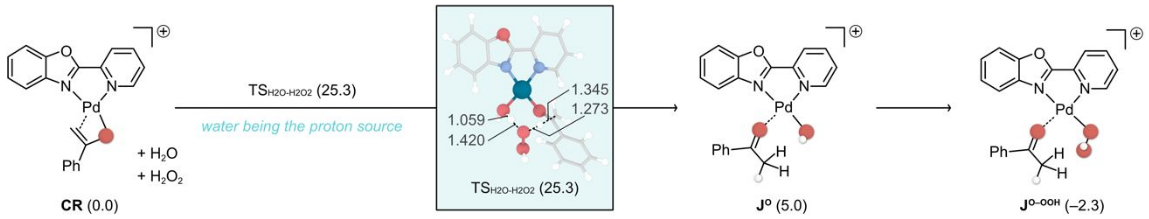

Scheme S2. Alternative protonation pathway from **CR** to form **J^{O-OOH}** with water being the proton source. Gibbs energies are reported at the experimental conditions in kcal/mol. The energies of the intermediates and transition states are referenced to the most stable intermediate **CR** (Figure 3a).

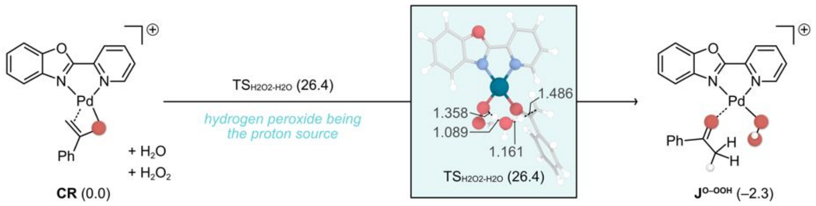

Scheme S3. Alternative protonation pathway from **CR** to form **J^{O-OOH}** with hydrogen peroxide being the proton source. Gibbs energies are reported at the experimental conditions in kcal/mol. The energies of the intermediates and transition states are referenced to the most stable intermediate **CR** (Figure 3a).

Natural bond orbital analysis of the Pd-enolate intermediate

Table S2. Selected NBO donor-acceptor interactions in **CR**, with the most relevant ones displayed in the inset of Figure 2. *BD* and *BD** denote bonding and antibonding orbitals, respectively. Second order perturbation energies (ΔE_{SOPT}) are given in kcal/mol.

Donor	*Orbital contributions*	*Acceptor*	*Orbital contributions*	ΔE_{SOPT}
BD (2)<br>O25–C29	74% O s (3%) p (97%)<br>26% C s (1%) p (99%)	BD* (1)<br>Pd24–C26	52% Pd s (6%) d (94%)<br>48% C s (10%) p (90%)	–23.30
BD (1)<br>Pd24–C26	48% Pd s (6%) d (94%)<br>52% C s (10%) p (90%)	BD* (2)<br>O25–C29	26% O s (3%) p (97%)<br>74% C s (1%) p (99 %)	–61.87

Microkinetic studies of the Wacker oxidation of styrene with H_2O_2 as the oxidant

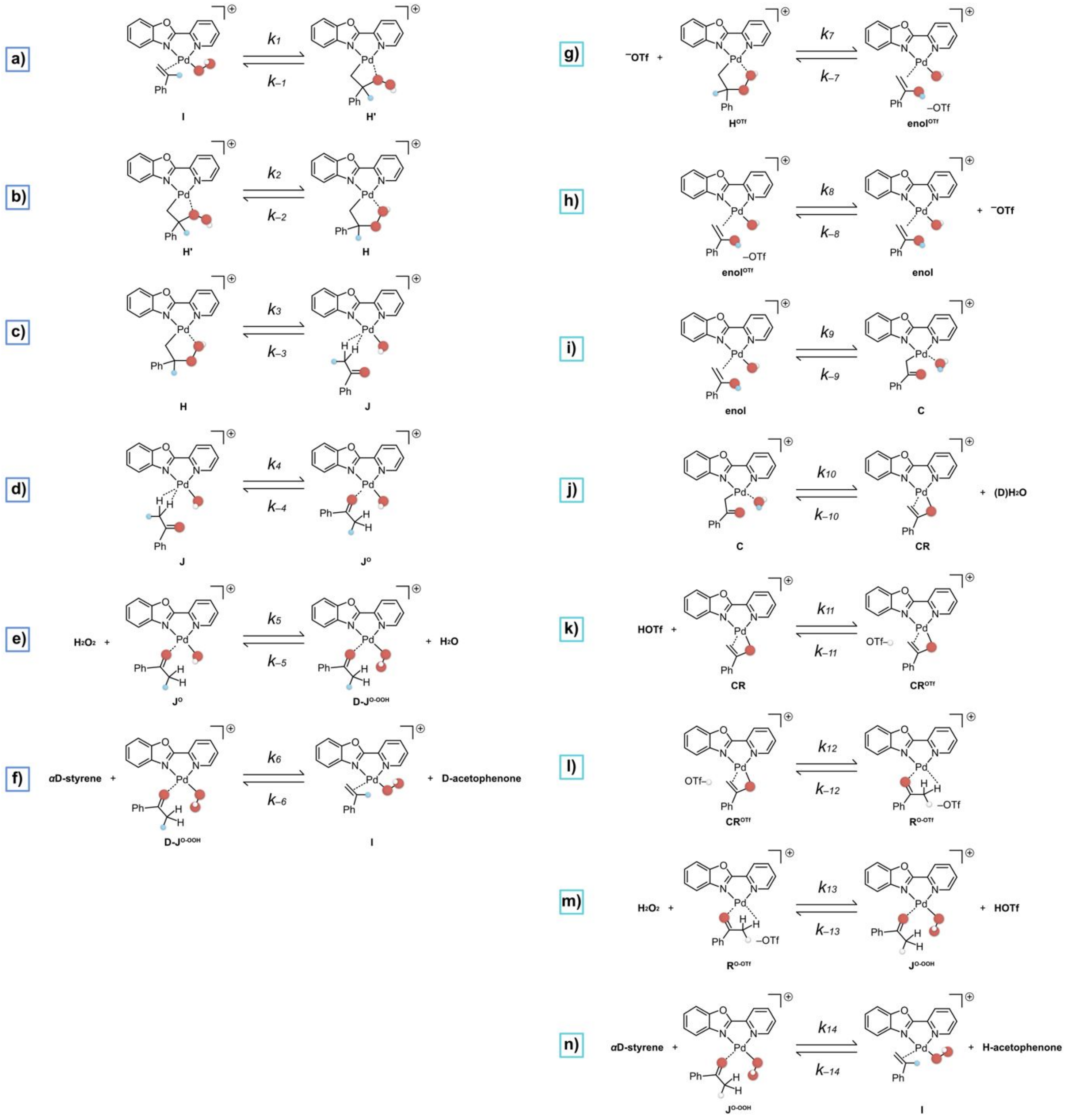

Scheme S4. Summary of the complete kinetic model considered for the Wacker oxidation catalytic cycle involving H_2O_2 as the oxidizing agent and α-D styrene as the substrate. The 1,2-hydride shift pathway (dark blue box), and the proton shuttle pathway (light blue box) are displayed.

The concentration *vs* time data were simulated (see Figure 3b) based on the kinetic model depicted in Scheme S4 and using the mass action reversible rate law as implemented in the software COPASI.[12] For all the elementary steps involving the association/dissociation of a molecule in Figures 1, 2 and 3a, we assumed an energy barrier of 5.0 kcal/mol. This value was chosen based on previous reports which showed that these processes are often diffusion limited with an estimated barrier of 5.0 kcal/mol.[13,14]

The DFT-calculated Gibbs reaction energies were used to compute the equilibrium ($K = k_1/k_{-1}$) and rate constants (k_1 and k_{-1}) according to the Arrhenius and Eyring equations shown below. The transmission coefficient (κ) in Eq. 2 was taken to be 1; this value assumes that every oscillation along the reaction coordinates takes the complex through the transition state.[15] The calculated rate constants are summarized in Table S3.

$$\Delta G^o = -RT\ln K \qquad (1)$$

$$k = \kappa \frac{k_B T}{h} e^{-\frac{\Delta G^\ddagger}{RT}} \qquad (2)$$

Table S3. Gibbs energies (in kcal/mol) and derived rate constants for the forward (k_1) and backward (k_{-1}) reactions at the experimental conditions: [**I**]$_0$ = 0.002 M, [styrene]$_0$ = 0.2 M, [H_2O_2] = 1.0 M, [H_2O] = 2.0 M. Rate constants for 1st and 2nd order reactions are given in units of s^{-1} or $Lmol^{-1}s^{-1}$, respectively.

	Rate constants	ΔG_{solv}
k_1	$2.99 \times 10^{+3}$	a) = 4.4
k_{-1}	$4.78 \times 10^{+6}$	
k_2	$2.45 \times 10^{+6}$	b) = –5.1
k_{-2}	$4.73 \times 10^{+2}$	
k_3	$1.13 \times 10^{+0}$	c) = –52.4
k_{-3}	7.90×10^{-39}	
k_4	$1.43 \times 10^{+9}$	d) = –21.9
k_{-4}	1.62×10^{-7}	
k_5	$1.43 \times 10^{+9}$	e) = –7.3
k_{-5}	$6.91 \times 10^{+3}$	
k_6	$1.43 \times 10^{+9}$	f) = –3.8
k_{-6}	$2.45 \times 10^{+6}$	
k_7	$1.40 \times 10^{+1}$	g) = –65.2
k_{-7}	4.67×10^{-47}	

	Rate constants	ΔG_{solv}
k_8	$7.56 \times 10^{+5}$	h) = 4.5
k_{-8}	$1.43 \times 10^{+9}$	
k_9	$1.43 \times 10^{+9}$	i) = –13.8
k_{-9}	1.28×10^{-1}	
k_{10}	$1.43 \times 10^{+9}$	j) = –4.0
k_{-10}	$1.75 \times 10^{+6}$	
k_{11}	$4.18 \times 10^{+3}$	k) = 7.6
k_{-11}	$1.43 \times 10^{+9}$	
k_{12}	$5.85 \times 10^{+3}$	l) = 2.2
k_{-12}	$2.34 \times 10^{+5}$	
k_{13}	$1.43 \times 10^{+9}$	m) = –12.1
k_{-13}	$2.21 \times 10^{+0}$	
k_{14}	$1.43 \times 10^{+9}$	n) = –3.8
k_{-14}	$2.45 \times 10^{+6}$	

Competing pathways for Wacker oxidation of styrene involving TBHP as the oxidant

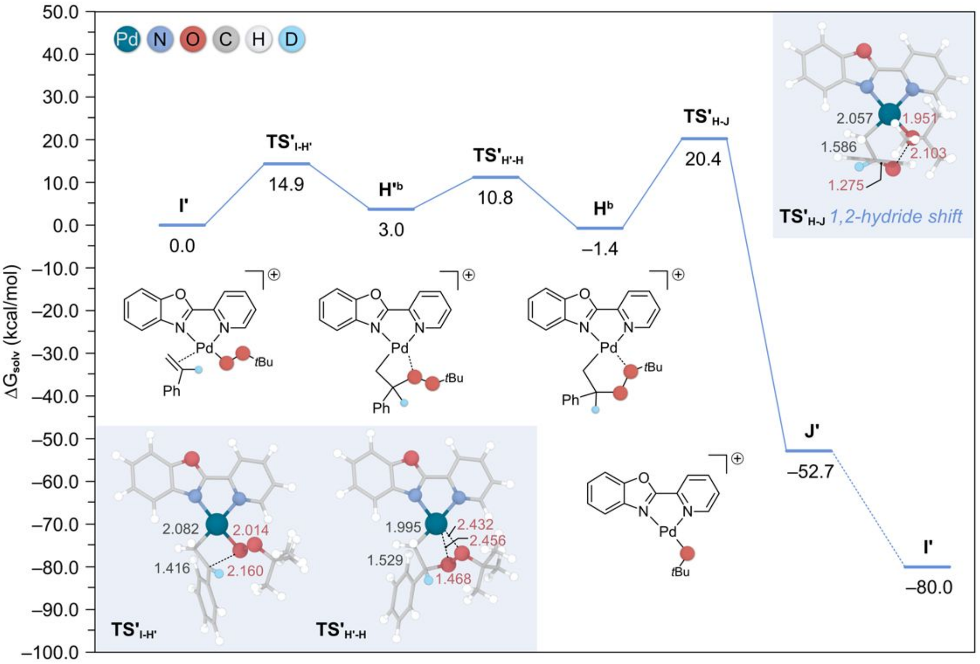

Figure S1. Gibbs energy profile reported in kcal/mol at 1 atm and 298.15 K for the 1,2-hydride shift pathway of Wacker oxidation catalytic cycle involving TBHP as oxidant and α-D styrene as the substrate. The optimized transition state structures are depicted as insets with the relevant bond distances shown in Å. For clarity, the deuterated alpha hydrogen is highlight as a light blue sphere.

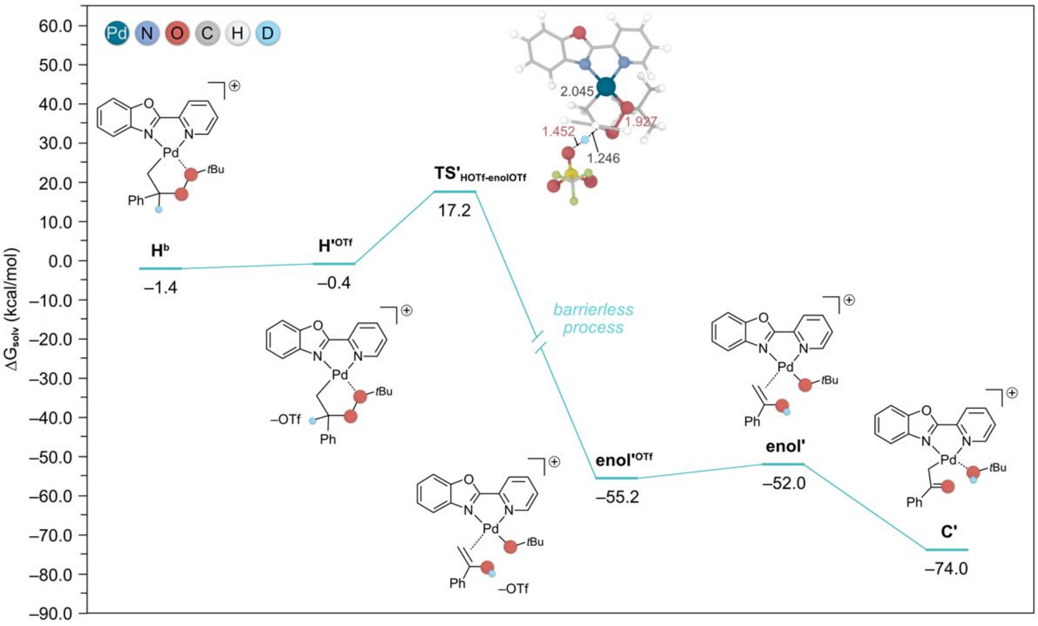

Figure S2. Gibbs energy profile reported in kcal/mol at 1 atm and 298.15 K for the proton shuttle pathway of Wacker oxidation catalytic cycle involving TBHP as oxidant and α-D styrene as the substrate, from the intermediate **H^{b}** to **C'**. The optimized transition state structures are depicted as insets with the relevant bond distances shown in Å. For clarity, the deuterated alpha hydrogen is highlight as a light blue sphere.

Microkinetic studies of the Wacker oxidation of styrene with TBHP as the oxidant

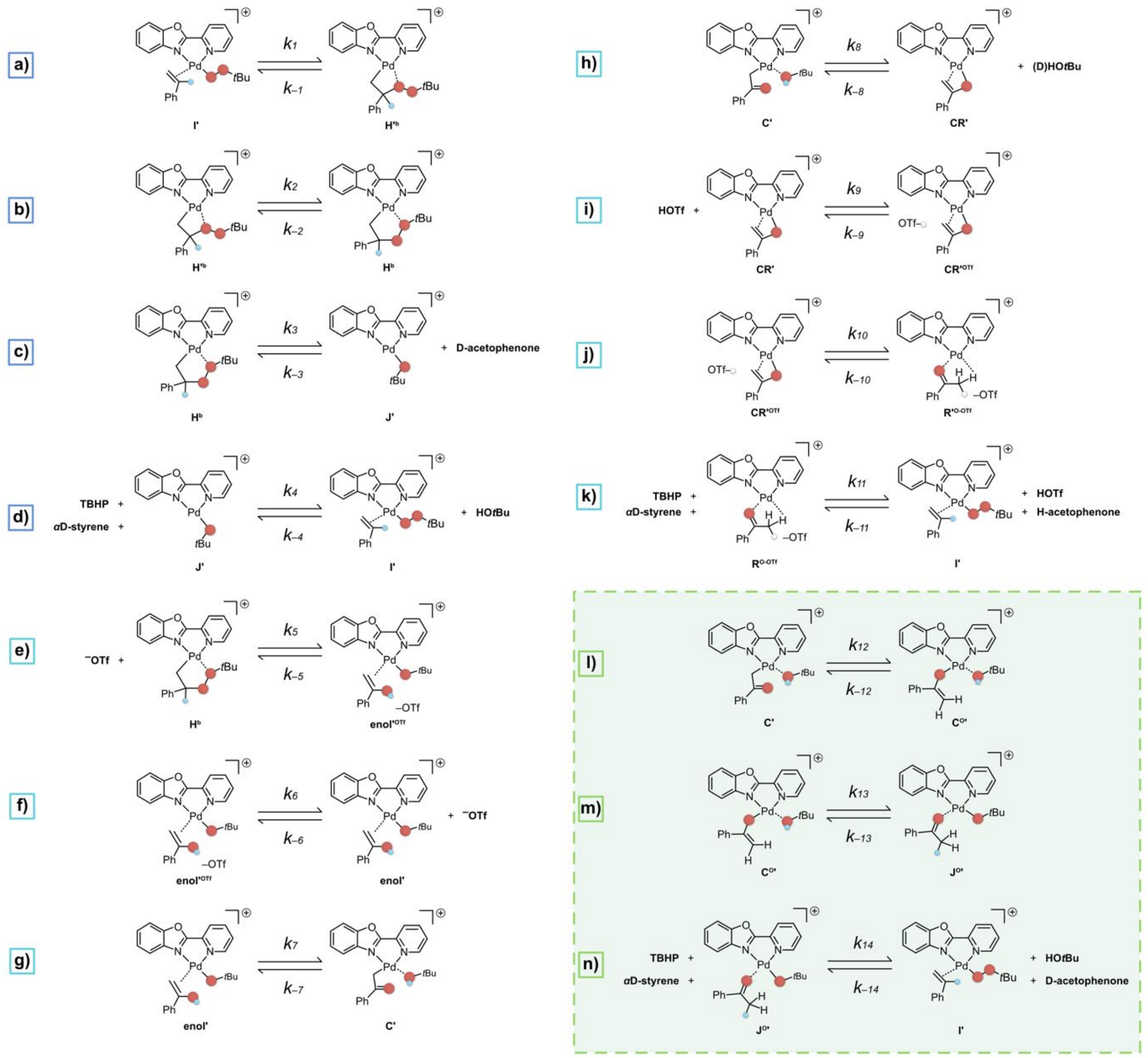

Scheme S5. Summary of the complete kinetic model considered for the Wacker oxidation catalytic cycle involving TBHP as oxidant and α-D styrene as the substrate. The 1,2-hydride shift pathway (dark blue box, Figure S1), the proton shuttle (light blue box, Figure S2 and Figure 4a) and the most favoured HO*t*Bu protonation (green box, Figure 4a) pathway are displayed.

The concentration *vs* time data were simulated (Figure 4b) based on the kinetic model depicted in Scheme S5 and using the same software as the H_2O_2 pathways mentioned in previous section. The rate constants were calculated as in previous, which are summarized in Table S4 reported in units of s^{-1} or $Lmol^{-1}s^{-1}$ for 1st and 2nd order kinetic reactions, respectively.

Table S4. Calculated Gibbs energies (in kcal/mol) and derived rate constants for the forward (k_1) and backward (k_{-1}) reactions at the experimental conditions: $[\mathbf{I}]_0$ = 0.006 M, $[\text{styrene}]_0$ = 0.2 M, [TBHP] = 2.4 M, [HOTf] = 0.006 M. Rate constants for 1st and 2nd order reactions are given in units of s^{-1} or $Lmol^{-1}s^{-1}$, respectively.

	**rate constants**	**ΔG_{solv}**
k_1	$8.84 \times 10^{+1}$	a) = 3.0
k_{-1}	$1.35 \times 10^{+4}$	
k_2	$1.31 \times 10^{+7}$	b) = –4.4
k_{-2}	$8.18 \times 10^{+3}$	
k_3	8.36×10^{-4}	c) = –51.3
k_{-3}	3.69×10^{-41}	
k_4	$1.43 \times 10^{+9}$	d) = –27.3
k_{-4}	1.89×10^{-11}	
k_5	1.79×10^{-1}	e) = –53.7
k_{-5}	1.41×10^{-40}	
k_6	$6.69 \times 10^{+6}$	f) = 3.2
k_{-6}	$1.43 \times 10^{+9}$	
k_7	$1.43 \times 10^{+9}$	g) = –22.0
k_{-7}	1.37×10^{-7}	

	**rate constants**	**ΔG_{solv}**
k_8	$3.58 \times 10^{+7}$	h) = 2.2
k_{-8}	$1.43 \times 10^{+9}$	
k_9	$4.18 \times 10^{+3}$	i) = 7.6
k_{-9}	$1.43 \times 10^{+9}$	
k_{10}	$5.85 \times 10^{+3}$	j) = 2.2
k_{-10}	$2.34 \times 10^{+5}$	
k_{11}	$1.43 \times 10^{+9}$	k) = –18.0
k_{-11}	1.12×10^{-4}	
k_{12}	3.06×10^{-4}	l) = 17.4
k_{-12}	$1.43 \times 10^{+9}$	
k_{13}	$6.18 \times 10^{+8}$	m) = –10.2
k_{-13}	$2.31 \times 10^{+1}$	
k_{14}	$1.43 \times 10^{+9}$	n) = –13.2
k_{-14}	3.50×10^{-1}	

Cartesian coordinates and energies of the modelled structures

All the calculated structures and corresponding energies can be found free of charge at the following open ioChem-BD repository: https://doi.org/10.19061/iochem-bd-6-314

References

(1) Chai, J.-D.; Head-Gordon, M. Long-range corrected hybrid density functionals with damped atom-atom dispersion corrections. *Phys. Chem. Chem. Phys.* **2008**, *10* (44), 6615–6620.

(2) Gaussian 09, Revision d1: Gaussian, Inc; Wallingford CT, 2016. https://gaussian.com (accessed 2023-12-21)

(3) Andrae, D.; Häußermann, U.; Dolg, M.; Stoll, H.; Preuß, H. Energy-Adjustedab Initio Pseudopotentials for the Second and Third Row Transition Elements. *Theor Chim Acta.* **1990**, *77* (2), 123–141.

(4) Ehlers, A. W.; Böhme, M.; Dapprich, S.; Gobbi, A.; Höllwarth, A.; Jonas, V.; Köhler, K. F.; Stegmann, R.; Veldkamp, A.; Frenking, G. A set of f-polarization functions for pseudo-potential basis sets of the transition metals Sc–Cu, Y–Ag and La–Au. *Chem. Phys. Lett.* **1993**, *208* (1-2), 111–114.

(5) Frisch, M. J.; Pople, J. A.; Binkley, J. S. Self-consistent molecular-orbital methods 25. Supplementary functions for Gaussian-basis sets. *J. Chem. Phys.* **1984**, *80* (7), 3265–3269.

(6) Binning, R. C.; Curtiss, L. A. Compact contracted basis sets for third-row atoms: Ga–Kr. *J. Comput. Chem.* **1990**, *11* (10), 1206–1216.

(7) McGrath, M. P.; Radom, L. Extension of Gaussian-1 (G1) theory to bromine-containing molecules. *J. Chem. Phys*. **1991**, *94* (1), 511–516.

(8) Curtiss, L. A.; McGrath, M. P.; Blaudeau, J. P.; Davis, N. E.; Binning, R. C.; Radom, L. Extension of Gaussian-2 theory to molecules containing third-row atoms Ga–Kr. *J. Chem. Phys.* **1995**, *103* (14), 6104–6113.

(9) Marenich, A. V.; Cramer, C. J.; Truhlar, D. G. Universal solvation model based on solute electron density and on a continuum model of the solvent defined by the bulk dielectric constant and atomic surface tensions. *J. Phys. Chem. B.* **2009**, *113* (18), 6378–6396.

(10) Walker, K. L.; Dornan, L. M.; Zare, R. N.; Waymouth, R. M.; Muldoon, M. J. Mechanism of catalytic oxidation of styrenes with hydrogen peroxide in the presence of cationic palladium(II) complexes. *J. Am. Chem. Soc.* **2017**, *139* (36), 12495–12503.

(11) *NBO 7.0.*: Theoretical Chemistry Institute; University of Wisconsin: Madison, 2018. https://nbo6.chem.wisc.edu (accessed 2023-12-21)

(12) Hoops, S.; Sahle, S.; Gauges, R.; Lee, C.; Pahle, J.; Simus, N.; Singhal, M.; Xu, L.; Mendes, P.; Kummer, U. COPASI—a COmplex PAthway Simulator. *Bioinformatics.* **2006**, *22* (24), 3067–74.

(13) McMullin, C. L.; Jover, J.; Harvey, J. N.; Fey, N. Accurate modelling of Pd(0) + PhX oxidative addition kinetics. *Dalton Trans.* **2010**, *39* (45), 10833–10836.

(14) McMullin, C. L.; Fey, N.; Harvey, J. N. Computed ligand effects on the oxidative addition of phenyl halides to phosphine supported palladium(0) catalysts. *Dalton Trans.* **2014**, *43* (36), 13545–13556.

(15) Atkins, P.; Paula, J.; Keeler, J. *Atkin's Physical Chemistry 11th ed*; Oxford, 2018. pp 793–794.
